# Correction: MACCHINI et al. Intra-Arrest Therapeutic Hypothermia and Neurologic Outcome in Patients Admitted After Out-of-Hospital Cardiac Arrest: A Post Hoc Analysis of the Princess Trial. *Brain Sci.* 2022, *12*, 1374

**DOI:** 10.3390/brainsci15070748

**Published:** 2025-07-14

**Authors:** Elisabetta MACCHINI, Emelie DILLENBECK, Martin JONSSON, Filippo ANNONI, Sune FORSBERG, Jacob HOLLENBERG, Anatolij TRUHLAR, Leif SVENSSON, Per NORDBERG, Fabio Silvio TACCONE

**Affiliations:** 1 Department of Intensive Care, Hôpital Universitaire de Bruxelles (HUB), 1070 Brussels, Belgium; elisabettamacchini@gmail.com (E.M.); filippo.annoni@erasme.ulb.ac.be (F.A.); 2 Department of Clinical Science and Education, Södersjukhuset, Centre for Resuscitation Science, Karolinska Institutet, 17177 Stockholm, Sweden; emelie.dillenbeck@regionstockholm.se (E.D.); martin.k.jonsson@ki.se (M.J.); sune.forsberg@tiohundra.se (S.F.); jacob.hollenberg@ki.se (J.H.); per.nordberg@ki.se (P.N.); 3 Emergency Medical Services of the Hradec Kralove Region, Department of Anaesthesiology and Intensive Care Medicine, Charles University in Prague, University Hospital Hradec Kralove, 50022 Hradec Kralove, Czech Republic; truhlaran@zzskhk.cz; 4 Department of Medicine, Karolinska Institute, 17177 Stockholm, Sweden; leif.svensson@ki.se; 5 Function Perioperative Medicine and Intensive Care, Karolinska University Hospital, 17177 Stockholm, Sweden

In the original publication [1], a wording error was introduced. Throughout the manuscript, Risk Ratio (RR) is used for comparisons. However, in the manuscript text, in Tables 3 and 4 and in the legends for Tables 3 and 4, the term Odds Ratio (OR) was mistakenly used instead of the correct Risk Ratio (RR). This error is limited to the terminology; the numerical values presented remain correct and correspond to RR. The only instance where Odds Ratio (OR) was actually used is in the random effects model, which is described in the manuscript but not included in the tables. In this context, Odds Ratio (OR) is the appropriate effect measure, and it is correctly reported. The results and conclusions of this study remain unaffected by this mistake.

The terminology has now been corrected in Table 3 and Table 4 and their legends, as shown below and in the manuscript. This correction was approved by the Academic Editor. The original publication has been updated.

**Table 3:** Primary outcome in the study population, which are presented as Risk ratios (RR) and 95% confidence intervals (CI). Data are presented in all patients and in the subgroup of patients with shockable (ventricular fibrillation or pulseless ventricular tachycardia) and non-shockable (asystole and pulseless electric activity) initial rhythm. CPC = Cerebral Performance Category.

Primary Outcome	Intervention	Control	Difference (95% CI)	Risk Ratio (95% CI)	*p*-Value
Survival with CPC 1–2 at 90 days, *n* (%)					
*All patients*	56/149 (37.6%)	45/142 (31.7%)	5.9 [−5.2–16.8]	1.19 [0.86–1.63]	0.29
*Shockable rhythm*	48/83 (57.8%)	35/78 (44.9%)	12.9 [−2.4–28.3]	1.29 [0.95–1.75]	0.10
*Non-shockable rhythm*	8/66 (12.1%)	10/64 (15.6%)	−3.3 [−15.3–8.6]	0.79 [0.33–1.87]	0.58

**Table 4:** Secondary outcomes in the population, which are presented as Risk ratios (RR) and 95% confidence intervals (CI). Data are presented in all patients and in the subgroup of patients with shockable (ventricular fibrillation or pulseless ventricular tachycardia) and non-shockable (asystole and pulseless electric activity) initial rhythm. CPC = Cerebral Performance Category.

Secondary Outcome	Intervention	Control	Difference (95% CI)	Risk Ratio (95% CI)	*p*-Value
Overall survival at 90 days, no (%)					
*All patients*	60/149 (40.3%)	52/142 (36.6%)	3.7 [−7.5–14.8]	1.10 [0.82–1.47]	0.09
*Shockable rhythm*	51/83 (61.4%)	41/78 (52.6%)	8.8 [−6.4–24.1]	1.17 [0.89–1.53]	0.25
*Non-shockable rhythm*	9/66 (13.6%)	11/64 (17.1%)	−3.5 [−15.8–9.1]	0.81 [0.36–1.81]	0.36
Survival with CPC 1 at 90 days, no (%)					
*All patients*	50/149 (33.6%)	35/142 (24.6%)	9.0 [−1.5–19.3]	1.36 [0.94–1.96]	0.11
*Shockable rhythm*	45/83 (54.2%)	27/78 (34.6%)	19.6 [4.6–34.6]	1.57 [1.09–2.25]	0.01
*Non-shockable rhythm*	5/66 (7.6%)	8/64 (12.5%)	−5.0 [−15.2–5.6]	0.62 [0.21–1.78]	0.60
